# Writing Technique Across Psychotherapies—From Traditional Expressive Writing to New Positive Psychology Interventions: A Narrative Review

**DOI:** 10.1007/s10879-021-09520-9

**Published:** 2021-09-14

**Authors:** Chiara Ruini, Cristina C. Mortara

**Affiliations:** Department of Psychology, University of Bologna, Viale Berti Pichat 5, 40127 Bologna, Italy

**Keywords:** Writing therapy, Journaling, Positive psychology, Psychotherapies, Emotion regulation, Autobiography, Expressive writing

## Abstract

Writing Therapy (WT) is defined as a process of investigation about personal thoughts and feelings using the act of writing as an instrument, with the aim of promoting self-healing and personal growth. WT has been integrated in specific psychotherapies with the aim of treating specific mental disorders (PTSD, depression, etc.). More recently, WT has been included in several Positive Interventions (PI) as a useful tool to promote psychological well-being. This narrative review was conducted by searching on scientific databases and analyzing essential studies, academic books and journal articles where writing therapy was applied. The aim of this review is to describe and summarize the use of WT across various psychotherapies, from the traditional applications as expressive writing, or guided autobiography, to the phenomenological-existential approach (Logotherapy) and, more recently, to the use of WT within Acceptance and Commitment Therapy (ACT). Finally, the novel applications of writing techniques from a positive psychology perspective will be analyzed. Accordingly, the applications of WT for promoting forgiveness, gratitude, wisdom and other positive dimensions will be illustrated. The results of this review show that WT yield therapeutic effects on symptoms and distress, but it also promotes psychological well-being. The use of writing can be a standalone treatment or it can be easily integrated as supplement in other therapeutic approaches. This review might help clinician and counsellors to apply the simple instrument of writing to promote insight, healing and well-being in clients, according to their specific clinical needs and therapeutic goals.

## Introduction

Writing therapy can be defined as the process in which the client uses writing as a means to express and reflect on oneself, whether self- generated or suggested by a therapist/researcher (Wright & Chung, 2001). It is characterized by the use of writing as a tool of healing and personal growth. From the first investigations of James Pennebaker (Pennebaker & Beall, 1986), writing therapy has shown therapeutic effects in the elaboration of traumatic events. In recent years, expressive and creative writing was found to have beneficial effects on physical and psychological health (Nicholls, 2009).

Currently, clinicians have moved from a distress-oriented approach to an educational approach, where writing is used to build personal identity and meaning through the use of autobiographical writing (Hunt, 2010). In this vein, autobiographical writing is becoming a widespread technique, which allows people to recall their life path and to better understand the present situation (McAdams, 2008). Moreover, it is observed that individuals often tend to report significant life events (positive or negative) in personal journals (Van Deurzen, 2012). Keeping a journal is a way of writing spontaneously: it can be considered a sort of logbook where thoughts, ideas, reflections, self-evaluation and self -assurances are recorded in a private way. Journaling is different from therapeutic writing the writer does not receive specific instructions on the contents and methodologies to be followed when writing, as it happens in therapeutic writing. Nowadays journaling can be done also through online blogs and social network (Facebook). In doing so, a private and spontaneous journal can be shared publically.

Writing techniques are often implemented into talking therapies, since both processes (talking and writing) favor the organization, acceptance and the integration of memories in the process of self-understanding (Lyubomirsky et al., 2006). However, expressive writing has been found to be beneficial also as a “stand alone” technique for the treatment of depressive, anxious and post-traumatic stress disorder symptoms (Reinhold et al., 2018). In a recent study, it was found that enhanced expressive writing (i.e., writing with scheduled contacts with a therapist) was as effective as traditional psychotherapy for the treatment of traumatized patients. Expressive writing without additional talking with a therapist was found to be only slightly inferior. Authors concluded that expressive writing could provide a useful tool to promote mental health with only a minimal contact with therapist (Gerger et al., 2021). Another recent investigation (Allen et al., 2020) highlighted that the beneficial effect of writing techniques may be moderated by individual differences, such as personality trait and dysfunctional attitude (i.e., high level of trait anxiety, avoidance and social inhibition). In these cases, therapeutic writing may be even more beneficial since it avoids the interactions with the therapist or other clients.

This article aims to illustrate and summarize the main psychotherapeutic interventions where writing therapy plays an important role in the healing process. For instance, a common application is the use of a diary in standard Cognitive Behavioral Therapy for promoting patients’ self observation (Butler et al., 2006). Similarly, other traditional psychotherapies use writing in their therapeutic process: from the pivotal application of writing to understand and overcome traumatic experiences, to the phenomenological-existential approach where writing has the function of giving meaning to events and of clarifying life goals (King, 2001), to Acceptance and Commitment Therapy, where writing facilitates the process of thought-defusion (Hayes, 2004). This review will also address the novel applications of writing technique to new a psychotherapeutic context: positive psychotherapy where the tool of writing is employed in many effective techniques (i.e. writing gratitude or forgiveness letters). Smyth (1998) reviewed 13 case-controlled writing therapy studies that showed the positive influence of writing techniques on psychological well-being. The benefits produced in writing activity (self regulation, clarifying life goals, gaining insight, finding meaning, getting a different point of view) can be described under the rubric of psychological and emotional well-being. In accordance with Fredrickson’s Broaden and Build Theory of Positive Emotions (2004), writing may foster positive emotions since putting feelings and thoughts into words widens scope of attention, opens up to different points of view and allows the mind to be more flexible (King, 2001).

Finally, we will describe and explore new contexts where writing activities currently take place: the web and social networks. We will underline important clinical implications for these new applications of writing activities.

## Traditional Applications of Writing Techniques

Clinical applications of writing therapy include the method of expressive writing created by Pennebaker (Pennebaker & Beall, 1986); the autobiography; and the use of a diary in traditional Cognitive Behavioral Therapy as described in the following sections.

### James Pennebaker: The Paradigm of Expressive Writing

James Pennebaker was the first researcher that studied therapeutic effects of writing. He developed a method called expressive writing, which consists of putting feelings and thoughts into written words in order to cope with traumatic events or situations that yield distress (Pennebaker & Chung, 2007). In the first writing project, Pennebaker and Beall (1986) asked fifty college students to write for fifteen minutes per day for four consecutive days. They were randomly instructed to write about traumatic topics or non-emotional topics. Results showed that writing about traumatic events was associated with fewer visits to the health center and improvements in physical and mental health. The experiment was repeated several times with different samples: with people who suffered from physical illnesses, such as arthritis and asthma, and from mental pathologies such as depression (Gortner et al., 2006). Individuals with different educational levels or writing skills were examined, but these variables were not found to be significant. At first, studies investigated only traumatic events, but later research expanded the focus to general emotional events or specific experiences (Pennebaker & Evans, 2014).

According to Pennebaker, what makes writing therapeutic is that the writer openly acknowledges and accepts their emotions, they become able to give voice to his/her blocked feelings and to construct a meaningful story.

Other therapeutic ingredients of expressive writing concern (1) the ability to make causal links among life events and (2) the increased introspective capacity. The former may be favored through the use of causality terms such as “because”, “cause”, “effect”; the latter through the use of insight words (“consider”, “know” etc.). These emotional and cognitive processes were analyzed through a computerized program (Pennebaker et al., 2015) and outcomes showed that the more patients used causation words, the more benefits they derived from the activity. Similarly, using certain causal terms expresses the level of cognitive elaboration of the event achieved by the patient and may indicate that the emotional experience has been analyzed and integrated (Pennebaker et al., 2003). Thus, the benefits of writing stem from the activity of making sense of an emotional event, the acquisition of insight about the event, the organization and integration of the upheaval in one’s life path.

Moreover, expressive writing allows a change in the way patients narrate life events. Many studies have highlighted that writing in first or third person alters the emotional tone of the narration (Seih et al., 2011). It is common for people who have experienced a severe traumatic event to initially narrate it in the third person and only later, once the elaboration and integration processes have set into motion, are they able to narrate their experience in the first person. This phenomenon occurs because third person narration allows the writer to feel safer and more detached from the experience, while first person perspective reminds them that they were the protagonist of the trauma. While writing using the third person can be easier in the wake of a traumatic event, writing in first person has been demonstrated to be more effective in the elaboration process (Slatcher & Pennebaker, 2006).

Another issue examined by Pennebaker and Evans (2014) concerns the difference between writing and talking about a trauma. In expressive writing, an important element consists of feeling completely honest and free to write anything, in a safe and private context without necessarily share the content with a listener or the therapist. Conversely, talking about trauma implies the presence of a listener, and the crucial aspect lies in the listener’s capability to comprehend and accept the patient’s narrative. Moreover, the interactions with a therapist could be particularly stressful for individuals with high levels of social inhibitions and trait anxiety (Allen et al., 2020).

### Writing Techniques for Addressing Trauma

Writing is considered a therapeutic strategy to cope with life adversities thanks to the positive effects of putting feelings and thoughts into words. There are various writing techniques used as therapeutic strategies to cope with a trauma which are described in Table 1.

**Table 1:** Writing approaches in clinical interventions

	Clinical sample	Theoretical model	Technique	Goal
Narrative exposure therapy	Post-traumatic stress disorder patients	Dual representation of traumatic memories (Elbert & Schauer, 2002)	Construction of a symbolic lifeline where stones and flowers represent negative and positive experiences. The clinician writes the autobiography and reads the narratives	–To decrease the avoidance through exposure –Elaboration of traumatic experiences –Development of hope (underling “flowers” moments) and resilience
Written Exposure Therapy	Post-traumatic stress disorder patients, veterans	Cognitive emotion-regulation (Martin & Dahlen, 2005)	In 5 weekly sessions patients write the trauma and describe the “hot spots” (moments of high emotional distress)	–To break the avoidance –“Hot spot” processing
Cognitive Processing Therapy	Post-traumatic Stress disorder patients, other diseases with intrusive symptoms	Cognitive–behavioral approach	After a psychoeducational phase, patients write sensory memories of trauma experience	–To decrease intrusive symptoms –To expose and process traumatic memories –To increase awareness
Logotherapy	Post-traumatic stress disorder patients; depression, anxiety	Phenomenological-existential approach	*Writing your epitaph*	–To express existential worries –To find meanings to events –To clarify values and goals
Acceptance and commitment therapy	Chronic illnesses, depression, anxiety	Cognitive–behavioral approach, relational frame theory	Thought defusion, *Writing your epitaph*	–To increase psychological flexibility –To promote active attitude

What the aforementioned writing therapies all have in common is a theoretical underpinning: the act of writing as a means to modify one’s life story and reframe elements which survivors want to change. Creating stories and thinking of ways to alter them may emphasize on one hand the possibility of a real change to occur, and on the other, the active role of the individual in their own life (Sandstrom & Cramer, 2003). In this way, writing can be also defined as a process of resilience: putting negative feelings into words can spark the search for solutions, with the consequence of having a positive attitude towards life challenges and promoting personal growth.

Besides the numerous positive effects of writing, there can be situations in which writing does not work, or when it can actually cause negative side effects. An example of said situation is when an individual has to deal with issues that arise intense painful emotions. In this case, writing can cause crying, very low mood, or even a breakdown (Pennebaker & Chung, 2007). This may occur because analyzing a traumatic experience may trigger a process of cognitive rumination, which is considered a specific symptom of PTSD (Pennebaker & Evans, 2014).

In conclusion, the paradigm of expressive writing is frequently used in patients who have had distressing experiences. Writing about traumatic experiences can help to elaborate negative emotions connected to the upheaval, to construct a narrative of the event, and to give it a meaning. However when client’s levels of distress are very intense and/or they are maintained by cognitive rumination, it is not advisable to undergo a writing exercise.

### McAdams: The Use of Autobiography in the Construction of Self-identity

Writing therapy has also been shown to have benefits in constructing self-identity (Cooper, 2014). An important pioneer of this method, Dan McAdams, developed a life story model of identity, which postulates that individuals create and tell evolving life narratives as a means to provide their lives with purpose and integrity (McAdams, 2008). Identity is an internalized story that is composed by many narrative elements such as setting, plot, character(s) and theme(s). In fact, human lives develop in time and space, they include a protagonist and many other characters, and they are shaped by various themes. Narrative identities allow one to reenact the past, become aware of the present and have a future perspective. Individuals construct stories to make sense of their existence, and these stories function to conciliate who they are, were and might be according to their self-conception and social identity. Biography, for example, is a written history of a person’s life; it deals with the reconstruction of a personal story in which salient events are selected and told. The therapeutic power of biographies entails the act of selection of worthy events that characterize a person’s life (Lichter et al., 1993).

In the same way, the autobiography can be an instrument to create a written life story. The first therapeutic effect is the possibility to define a sense of identity through autobiographical narratives by the identification of significant personal changes and by giving meaning to them. According to Bruner (2004) writing an autobiography allows the clients to recognize themselves as the authors of their experiences (sense of personal agency).

Another therapeutic ingredient of autobiography is the process of conferring stability to autobiographical memories: people often misremember details of events over time or are influenced by distortion mechanisms (McAdams, 2008). Autobiography is useful not only to code every event of self-story, but also it is beneficial for integrating different experiences and for analyzing the life trail, highlighting both continuity and changes. McAdams studied the use of autobiography in life changes, by employing a written procedure, the “Guided Autobiography” (McAdams et al., 2006). This is a therapeutic technique aimed at investigating the relationship between the continuity of story themes and personality changes. In the span of ten two-hour sessions, which take place once a week, participants are asked to think and describe the most important events of their life, referring to a specific life theme (i.e. family, money, work, health, spirituality, death, aspirations). Reker et al. (2014) underlined that Guided Autobiography is an effective method to enable participants to understand and appreciate their life stories, which also increases optimism and self-esteem. In conclusion, Mc Adams technique of guided autobiography entails different therapeutic ingredients: it allows to connect life events and personal memories, and to underscore the process of continuity among them. At the same time, significant life changes are emphasized, and the individual can improve the sense of agency in understanding his/her role as a protagonist of his/her life. Thus, guided autobiography could enhance personal well-being and meaning in life.

### The Use of the Diary in CBT

Considering that Cognitive-Behavioral Therapy (CBT) works on thinking patterns, maladaptive thoughts and dysfunctional behaviors (Butler et al., 2006), the diary is a very useful instrument of self-observation. It entails a written exercise in which the client is asked to take note of when and where a stressful situation occurs, the automatic thoughts it elicits, the connected emotions and the consequent behavior. This technique was developed by Aaron Beck at the early stages of cognitive therapy for anxiety and depression (Beck, 1979). In writing this diary the writers learn to pay attention to their functioning and acquires self-awareness about their problematic issues (King & Boswell, 2019). According to traditional CBT method, the therapeutic ingredients of writing the structured diary consist of helping clients to increase their awareness of automatic thoughts and beliefs, which are influencing their emotions and behaviors. The diary then allows the processes of cognitive restructuring, where negative, automatic thoughts are analyzed and modified in order to achieve a more realistic attitude toward life events and problematic situations (Beck, 1979). Thus, writing techniques within CBT consist of keeping a structured diary, which is supervised by the therapists along the various phases of the therapeutic process. The diary in CBT is specifically aimed at addressing symptoms and distress, but it can also trigger cognitive changes, maturation and improved self-awareness at the end of the clinical work (Butler et al., 2006).

## Existential Approaches: The Bridge Between Clinical Psychology and Positive Psychology

### Logotherapy

Logotherapy is a specific strategy within phenomenological-existential therapies. It relies on a therapeutic paradigm created by Viktor Frankl (Frankl, 1969) and based on existential issues. Logotherapy is an entirely word-based treatment. Its tenets assume that life always has meaning, even in the most adverse circumstances and that people always strive to find a personal meaning in their existence (assumption of will to meaning). From this perspective, a journal can be considered a place where people find a meaning in life-threatening events and transform implicit and negative experiences into expressive and positive ones. In fact, Logotherapy emphasizes the importance of words in creating a meaning and, in this case, writing techniques are particularly appropriated to this task. The client is asked to narrate adverse life events using words and sentences that help him/her to acquire a sense of meaning and acceptance.

In phenomenological-existentialist psychotherapies, writing assignments are used to increase clients’ awareness of their limitations and to create an opportunity to reflect on both life and death (Yalom, 1980). Specifically, in the exercise of *Writing your Epitaph,* the client is encouraged to think and write what people would say in their memory. This task aims at clarifying personal values and at committing to them. This allows the identification of the direction individuals want to give to theirs life and to verify if they really are acting towards those goals. The main difference between logotherapy and guided autobiography relies on the philosophical framework used in existential approach, which is not present in Mc Adams paradigm. Furthermore, in logotherapy the narrative topic might be narrowed to a specific traumatic event, not necessarily involving all personal biography. The therapeutic ingredients of logotherapy, thus, concern the increase in life meaning and the possibility of reframing and processing existential issues as death, evil and trauma in individual’s life experiences.

### Acceptance and Commitment Therapy

Acceptance and Commitment Therapy (ACT) focuses on the acceptance of unchangeable things and on the integration of different interventions (including strategies of mindfulness) with the aim of increasing psychological flexibility and promoting an active attitude towards problematic matters (Hayes, 2004). ACT is based on a rigorous cognitive analysis, the Relational Frame Theory (Reese, 2013). This theoretical frame posits that language and cognition allow humans to have the ability to learn to relate events under arbitrary contextual control. This framework particularly analyzes paradoxes, metaphors, stories, exercises, behavioral tasks, and experiential processes (Hayes, 2004). This approach has studied a particular mechanism called “Thought Defusion” (Hayes, 2004) which deals with the ability to distance one’s self from problematic thoughts. Frequently, individuals cannot see problems because they are “fused” with them. The defusion techniques allow the individual to distance themselves from problems and see them from a more detached perspective (i.e., the helicopter perspective exercise). In this way, patients have the possibility to identify a problematic issue, accept it and find a manner to live with it, which can decrease the level of suffering (Hayes, 2004). Thus, the act of writing can be considered as a way to keep distance from one’s own thoughts and feelings in order to be able to modify the behaviors and life choices according to one’s values and priorities.

The first part of this article identifies how the traditional use of writing techniques has been analyzed within different forms of psychotherapy. The subsequent part of this review will describe the application of similar writing techniques within the framework of positive psychology. In particular, the use of expressive writing, journaling or other structured writing techniques will be described as ways to promote personal well-being, personal growth, gratitude and positive emotions in general.

## Positive Psychotherapy

Unlike the traditional deficit-oriented approach to psychotherapy, Positive Psychotherapy aims at considering with a similar standing, symptoms and strengths (Rashid & Seligman, 2018).

Positive Psychotherapy uses writing techniques in various moments of the therapeutic process (see Table 2). For instance, at the beginning of the therapy clients are invited to write a personal presentation in positive terms. This exercise is called “*Positive Introduction”* (Rashid, 2015). The importance of this exercise lies in the fact that while writing a self-presentation clients highlight their positive characteristics and qualities and they may also recall and describe a particular episode when these strengths were manifested. This initial writing assignment, thus, may foster patients’ self-esteem and self-awareness of positive personal characteristics. In the middle phase of the positive psychotherapy, therapists can suggest the “*Positive Appraisal”* activity to their clients. This consists of thinking and writing down resentments, bad memories and negative events which have occurred in their past and that still affect their life. Clients are asked to reframe these past negative events and to search for possible positive consequences in terms of meaning or personal development. The final phase of therapy focuses on exploring and training the individual’s strengths. The exercises proposed in this phase include writing assignments such as “*Gift of Time”* and “*Positive legacy”,* where the therapist asks clients to write how they would be remembered by significant others and future generations. “*Positive Legacy”* is focused on the positive connotations of writing and often it is associated with planning a “gift of time activity” that puts these positive characteristics into practice (Rashid, 2015). This technique entails similarities with logotherapy and ACT epitaph exercise, but in PPT the client is guided to emphasize positive aspects of their life and personal qualities, and there is no mention to relational frame theory as in ACT.

**Table 2:** Writing approaches in positive intervention

	Use	Theoretical model	Techniques	Goal
Gratitude	Depression, anxiety	Positive psychotherapy	–Gratitude letters –Gratitude journal –Good versus bad memories	–To strengthen relationships –To enhance well-being
Forgiveness	Clients who express feeling of anger or bitterness	Positive psychotherapy	Forgiveness letters	–To promote emotional regulation –To spark positive attitude towards others
Wisdom	Posttraumatic embitterment disorder	Positive psychotherapy, Narrative Therapy	–Guided autobiography –Use of fairytales	–To spark self-awareness and self-acceptance –To integrate life experiences –To promote different perspectives
Hope	Post-traumatic stress disorder patients, chronic illnesses	Positive psychotherapy	–Writing about best possible selves –Blessing journal/three good things	–To provide positive emotions –To enlarge perspectives

Furthermore, Positive Psychotherapy entails also specific writing techniques devoted to the promotion of specific positive emotions, such as gratitude, forgiveness and wisdom, as described below.

### Gratitude

Gratitude is a feeling of appreciation for people or events, which is triggered by the perception of having obtained something beneficial from someone or something (it can be also an impersonal source, such as God or Nature) (Rashid & Seligman, 2018). Written exercises of gratitude can be divided into *Gratitude Letter, Gratitude Journaling* and “*Good versus Bad Memories”*.

*Gratitude Letter* consists of writing and delivering a gratitude letter to a person that the client has never sincerely thanked. This intervention aims at strengthening the client’s relationships and enhancing their social well-being (Lambert et al., 2010). In *Gratitude** Journaling,* clients are asked to write three good things which have happened to them during the day (Rashid, 2015).

Many studies showed that thinking about memories of gratitude in a written form promotes well-being and increases positive mood because writing allows one to give shape to positive experiences (Toepfer et al., 2012; Wong et al., 2018). In fact, in gratitude writings individuals are more likely to express positive feelings and have high level of insight, making gratitude letters or journaling a powerful tool to produce not only well-being, but also health improvements (Jans-Beken et al., 2020).

Difficulties in writing a gratitude letter relate to the interpersonal nature of this task, because being grateful towards someone entails being dependent on that person and, in turn, this can invoke a sense of vulnerability that makes the writer feel not at ease (Kaczmarek et al., 2015). In this way, the psychological costs of writing a gratitude letter are greater than expressing it in a private journal. Another important element of difference pertains the delivery of the letter as the gratitude journal has a personal use, while the letter is written to be delivered to someone. The main risk of writing a letter to someone refers to the possibility of not being accepted or feeling judged by the reader. For this reason, recently, positive therapists may ask their clients to write the letter, without necessarily have it delivered to the recipient. Thus, the benefits associated with a gratitude letter exercise are not necessarily connected with the act of delivery, but are placed in the writing itself (Rash et al., 2011).

In addition to gratitude letter and journal, *Good versus Bad Memories* is a writing activity which has the therapeutic effect of helping clients to understand how anger, bitterness and other depressive symptoms may influence clients’ life and how they can stop these processes by focusing on positive memories and experiences (Rashid & Seligman, 2018).

These three writing activities on gratitude are useful in order to emphasize good things that usually are taken for granted. Furthermore, they may downregulate the impact of negative emotions or negative experience in life. (Lyubomirsky & Layous, 2013).

### Forgiveness

Forgiveness implies a situation of offense where a person makes the choice of letting go of anger and of searching for a compassionate attitude towards the transgressor (Thoresen et al., 2008).

Evidence shows that writing about an interpersonal conflict can decrease the level of negative effects in relational conflicts (Gordon et al., 2004). The act of writing a forgiveness letter includes a cognitive processing that promotes emotional regulation, the expression of affect and gaining insight. Positive Psychotherapy uses forgiveness exercises in order to transform feelings of anger and bitterness into neutral or positive emotions (Rashid, 2015). For example, clients are asked to write a letter where they describe an experience of offence with related feelings and then the promise to forgive the guilty person. McCullough et al. (2006) found that victims of interpersonal transgressions could became more forgiving toward their transgressors when they were asked to write about possible beneficial effect of the transgression, compared with victims who wrote about traumatic or neutral topics. Thus, the positive narrative approach may facilitate forgiveness and help victims to overcome traumatic interpersonal issues.

As for gratitude letters, the delivery of forgiveness letters is a crucial issue, because the act of showing forgiveness can influence the process of forgiveness itself. In some cases, as highlighted in Gordon and collaborators’ study (2004) about marital conflicts, writing and delivering a letter is helpful to reduce relational tension and the consequent conflicts, but in other situations where forgiveness remains an intra-personal process, sharing it can be more harmful than beneficial. This may occur particularly when the relationship between victims and transgressors is particularly problematic (or even abusive) and reconciliation is not possible, or not recommendable (Gordon et al., 2004).

Forgiveness writing is also helpful in the promotion of self-forgiveness. Jacinto and Edwards (2011) describe a case where the exercise of writing a letter was used in the therapeutic process of self-forgiveness. The act of writing helped the client to trigger self-empathy and consequentially to let go of negative beliefs about herself.

In conclusions, the therapeutic ingredients of these writing assignments (gratitude and forgiveness letters) concern both an intra-personal dimension (the promotion of self-esteem, self-awareness and a sense of meaning in life) and an interpersonal dimension (the promotion of empathy, compassion, and a sense of connectedness with others). They both constitute the pillars of well-being and positive psychological functioning (Lyubomirsky & Layous, 2013).

### Wisdom

Wisdom is a complex ability composed of cognitive and emotional competences, such as perspective-taking, thinking with a long-term perspective, empathy, perception and acceptance of emotions (Staudinger, 2008). Collecting narratives of wisdom may be connected with autobiographical memory (McAdams, 2008). Glück and collaborators (2005) conducted a study where participants had to write a 15 line paragraph describing all the situations where they did, thought or said something wise; then they had to select a situation from them where they had been wise. This writing task was followed by an interview in which the “wise situations” were discussed. Writing about autobiographical memory kindles the development of strengths related to wisdom, such as acceptance and forgiveness of others, taking different perspectives, being honest and responsible and making compromises.

The promotion of wisdom can be done also using specific narrative structures, such as the one of storytelling and fairytales. The employment of fairytales with adults was found to promote the development of feelings of wisdom (Ruini, 2014; Ruini & Ottolini, 2014). Fairytales, in fact, enable a process that allows one to reformulate problems in narrative terms, using a specific narrative plot. It consists of three main steps: (1) the identification of an initial stressful event; (2) the journey of the protagonist, with tests and adversities to face; (3) the final positive resolution (happy ending) (Masoni, 2019). Furthermore, Ruini and Ottolini (2014) showed the effectiveness of using fairytales in patients who had to cope with life transitions: to read and then to re-write a fairytale is a way to symbolize one’s own life and clarify moral and existential issues. In this particular narrative technique, the patients are asked to write a fairytale that well symbolizes their life, with a happy ending; the fairytale created is then read and discussed in the session. In many cases, the stories contain narrative issues and characters’ attitudes that evidence patients’ real difficulties. For example, the protagonist can be very passive in the story or not well characterized. The clinician helps the patient to re-write the fairytale making corrections that allow the patient to clarify dysfunctional elements and consequently to construct a new and more positive story. Creating a happy ending can be a way to express patients’ desires about their future and to let them imagine how they can be satisfied with their life. Through the exercise of writing a structured fiction story, patients can analyze their life situations in a more detached way, view problems from a different perspective and become aware of their values and attitude. This emotional detachment is similar to the process of cognitive defusion in ACT, but in this case, there is no mention to the relational frame theory, and the act of writing the fairytales may promote another positive dimension: creativity (Ruini et al., 2020).

### Hope

Writing can be considered as a coping strategy aimed at finding solutions which in turn can spark hope in desperate situations. In line with the expressive writing approach (Pennebaker & Evans, 2014), writing about traumatic or particularly painful situations, may promote feelings of hope because it allows to go beyond suffering and to reach positive perspectives.

Positive psychotherapy entails specific written exercises that focus on strengthening hope. Among these, we can find *Writing about Best Possible Selves,* where possible selves are personal representation of goals, connected with what people desire for their future. This writing exercise aims to improve self regulation because it allows clients to clarify and restructure priorities and acquire insight on one’s own motivations and values (Loveday et al., 2018; Sheldon & Lyubomirsky, 2006). Other potential benefits of writing “best possible selves” are the possibility of integrating life experiences, identifying the meaningful ones, and gaining a sense of control (Lyubomirsky & Layous, 2013). These benefits have also been confirmed in King’s (2001) study where participants were invited to write for four days (20 min per day) a narrative description of their best possible future selves. Outcomes showed that, compared to writing about other topics, the act of thinking and describing oneself as best as possible increased positive mood and decreased distress five months later.

Another technique to foster hope is the *Blessing journal/Three Good Things* in which patients keep a journal where they write three good things every night and the reason why they think those things have happened. The objective is not only to identify positive happenings, but also to search for the causes and underline the active role of the subject in provoking them. The sense of personal agency in fact, is considered a component of hope (Snyder et al., 2000). Snyder’ hope therapy (Snyder et al., 2002) entails the specific use of “hope narratives” where clients are guided in a process of writing past experiences where they were able to achieve significant personal goals (hope reminding exercise). In the subsequent phase of hope therapy, clients are asked to write specific narrative where they focus on future goals to be achieved and they develop specific path to reach them (hope building techniques).

In conclusion, the traditional use of writing techniques within psychotherapies has been included also in the positive psychology perspective. However, positive psychotherapy and other positive interventions have changed the focus of the writing exercises from negative/traumatic experiences to positive ones (Lyubomirsky et al., 2006). The therapeutic process may be the same, but the focus is shifted from symptoms to well-being. The positive psychology approach promotes writing exercises on gratitude, hope, forgiveness, and positive descriptions of oneself with the intention of improving clients’ well-being. At the same time, these exercises may help them to process also negative emotions and traumatic events (McCullough et al., 2006). Conversely, in traditional psychotherapies (as described in the first part of this article) writing techniques are specifically aimed at overcoming negative events and psychological symptoms. As a byproduct, they may also favor patients’ recovery, well-being and meaning in life, but this was not their main therapeutic purpose.

## New Applications: Writing on the Web

The final part of this review deals with other recent modifications of writing approaches that entail the use of internet and other digital technologies. In recent years the act of journaling and keeping a diary has been often replaced with writing in blogs or on social networks such as Facebook, Twitter or Instagram. On these platforms users may create personal profiles that reflect their sense of identity or positive introduction. User profiles also include the narration of their meaningful experiences via photos, videos or boards. In this section we will describe these phenomena and their relation with the psychotherapy process, underlying both the positive and negative features.

Writing activity has been recently modified by the use of the web and by the influence of technological instruments. In fact, social networking sites constitute a technological tool for self-revelation which gives the opportunity to share experiences and impressions through writing as they can create a permanent record of one’s actions or thoughts (Sauter, 2013). In social-networks the personal home page is both a space of identity construction and of self presentation towards the rest of the world (Sorapure, 2003). In particular, Facebook can also be considered an online autobiographical instrument that codes and keeps track of events of in one’s life.

In this way, unlike traditional handwriting, writing on the web implicates a social function rather than a private one. Sharing, in fact, is a fundamental component of self-writing on the web.

Additionally, the presence of clinicians in social networks is a relevant factor: some therapists can use social networks as an extra instrument to give support or be available for patients. Taylor et al. (2010) observed that the client–psychotherapist relationship can be influenced by the presence of the psychologist on the Internet. Many psychotherapists create a web site where clients can find their professional activities and services. Other psychologists let clients contact them via e-mail or instant messaging when they need help (Manfrida et al., 2017). For example, “Talkspace” is a web platform which offers online therapy through messaging with a licensed therapist. Hull and Mahan (2017) studied the effectiveness of Talkspace’s text-based therapy and showed the beneficial effects that text therapy had on symptom reduction and improvement in psychological well-being. The study participants also reported high levels of satisfaction with the treatment.

Moreover, Sloan et al. (2015) have studied the efficacy of a structured writing therapy conducted via internet: “Interapy”. It consists of a protocol of 10 writing sessions, held twice a week, in which patients who suffered from post-traumatic stress disorder had to write about the traumatic event focusing on cognitive reappraisal and sharing details with someone close. A trained therapist then gave feedback on every written online narrative.

Recently Botella et al. (2017) presented a new instrument, which utilizes digital technology and virtual reality via web in a framework of positive interventions. They named it “Book of Life” and it consists of a personal digital diary composed of various chapters in which some narrative exercises are proposed. Participants may also include multimedia contents (i.e. pictures, videos, music) about a specific topic, in order to create a final positive autobiographical narrative. The therapeutic aim of the Book of Life is to foster positive emotions and the use of personal psychological resources. The results of clinical trials where the Book of Life was applied were particularly effective with older adults and cancer patients.

In conclusion, writing on the web involves different types of interventions. These techniques appeared to be particularly useful during the lockdown due to the Covid-19 pandemic. The use of online platforms and the possibility to contact therapists via mail or social network allowed the delivery of mental health treatments during a global stressful experience (González-Robles et al., 2021). Future investigations are needed to explore how writing in websites and social networks may influence the development and the delivery of psychological therapies, both the traditional and the positive ones.

## Conclusions

Considering the various and different applications, writing therapy constitutes a very adaptable technique to be used as a standalone treatment or as a supplement of other therapies. WT may provide beneficial effects on symptoms and also on psychological well-being. In fact, the act of writing showed great potential in the promotion of personal strengths, resilience and post-traumatic growth (Sandstrom & Cramer, 2003). Moreover, writing techniques can be considered a tool of continuity from the traditional approaches to the new psychotherapy contexts, such as positive psychology and the employment of digital technologies in psychotherapeutic settings.

In general, although it is common sense to think that psychotherapy is for the majority orally communicated, the act of writing provides many benefits in psychotherapeutic sessions as well as in clients’ daily life. Writing makes thoughts more real and transforms mental states in something concrete as feelings, whereas thoughts and reflections expressed orally can easily disappear when the psychotherapy session ends. Moreover, writing therapy may be particularly effective for individuals with high levels of interpersonal avoidance or social inhibition, since they have a therapeutic tool for managing their difficult emotions, without the burden of a direct interaction with the therapist (Allen et al., 2020).

However, some caution is needed also when applying writing techniques within psychological interventions. First, the use of writing technique may give thoughts more emphasis and power, specifically when writing negative thoughts or feelings. In this case, the act of writing may increase cognitive rumination (Pennebaker & Evans, 2014). The same potentially negative effect of writing thoughts and life experiences may apply also to positive issues. Lyubomirsky et al. (2006) found that individuals who were asked to write about their happiest moments experienced reduced well-being. The author suggested that the analytic nature of writing about positive events may be counterproductive as opposed to the unorganized process of simply thinking about them.

Another possible side effect of writing techniques (both documented for traditional psychotherapies and positive interventions) is the sense of shame that can be triggered when someone else reads the writing. This could imply a resistance to the act of putting down negative thoughts or the worsening of worries because of the excessive interpersonal exposure (Pennebaker & Evans, 2014). In these cases, it is recommended that the use of writing may remain confidential, or it may be accompanied by specific psychological support, so that negative emotions can be discussed with the therapist.

Similar issues of shame and embarrassment have been found to occur when writing and delivering gratitude letters: some studies (Boehm et al., 2011; Layous et al., 2013; Shin et al., 2020) underlined the specific influence of cultural issues: in collectivist cultures (vs individualistic ones) expressing gratitude resulted less effective on well-being because of the sense of indebtedness and embarrassment it can provoke. In fact, the sense of self-improvement and personal agency, which were emphasized in writing assignments, increased life satisfaction only in Western individuals. Conversely, collectivist cultures consider self-focus and individual goals in a less positive way, since they may interfere with the need of the group. Thus, when working with clients belonging to collectivistic cultures, clinicians should use caution in prescribing writing assignments (such as gratitude and forgiveness letters) that can interfere with clients’ relationships within the community they belong to. However, the beneficial effect of writing can be preserved, if the delivery of the letter is not mandatory (Lyubomirsky et al., 2006; Pennebaker & Evans, 2014).

In conclusions, this review of the literature briefly described writing techniques within psychological therapies, that encompass several different methodologies and specific exercises, ranging from unstructured journaling to personal autobiography, to recalling specific memories associated with positive and/or negative experiences, to writing fairytales, short stories, or letters of forgiveness and gratitude, etc. All these methodologies could be easily implemented in many psychotherapeutic approaches, from the traditional CBT, logotherapy and existential therapies to novel approaches, such as positive interventions. Although some authors found certain potential side effects of writing techniques on the emotional well-being of patients (Lyubomirsky et al., 2006), a large body of literature confirmed their beneficial effects, which amplify and prolong the therapeutic effect of the talking therapy with the clinicians. Importantly, adding writing techniques to talking therapies was found to reduce the length of treatment and improve access to psychological therapies (Gerger et al., 2021; Pennebaker, 2018). The integration of writing techniques within traditional talking therapies or new positive interventions could be easily done also using technological devices, such as app, emails, on line journals or social networks (Botella et al., 2017) which could be more appealing for younger patients. The technological tools of communication are changing the role of therapists, who more often use on line resources to support their clinical work. Furthermore, the pandemic due to the Covid 19 and the need to implement telepsychology and distant mental health interventions make the integration of writing techniques particularly appropriate in these settings.

A final recommendation concerns the selection of writing exercises and the timing to prescribe them during the therapeutic process. Different writing activities could yield different effects according to patients’ clinical status and emotional balance. Certain activities could be used to deal with specific clinical problems, such as overcoming traumatic events or personal transgressions, and should be prescribed when the patient complains some of these issues. Other writing activities, on the other hand, have an unspecified theme, (i.e., guided autobiography, or writing your own epitaph) and they appear to be more appropriate for promoting personal growth, personal awareness and existential well-being. These activities may be well suited for the concluding part of the psychological treatment, independently of patient’s initial symptoms and problems. Research on positive interventions (Lyubomirsky & Layous, 2013) documented the need to consider the extent to which the therapeutic activity matches an individual’s preference and characteristics (i.e., “person × intervention fit”) in order to maximize the beneficial effect of the intervention on happiness and well-being. Similarly, Joseph (2015) suggested that therapists should follow their clients preference and should co-create with them a specific treatment agenda, unique for that client, instead of referring to a set of pre-determined list of activities (in this case writing assignments). This would be a more flexible and creative therapeutic approach, in line with a positive clinical psychology perspective (Ruini, 2017). However, only further clinical research should test and verify the most effective approach in prescribing writing assignments during the course of psychological interventions.

## References

[CR1] Allen SF, Wetherell MA, Smith MA (2020). Online writing about positive life experiences reduces depression and perceived stress reactivity in socially inhibited individuals. Psychiatry Research.

[CR2] Beck AT (1979). Cognitive therapy of depression.

[CR3] Boehm JK, Lyubomirsky S, Sheldon KM (2011). A longitudinal experimental study comparing the effectiveness of happiness-enhancing strategies in Anglo Americans and Asian Americans. Cognition & Emotion.

[CR4] Botella C, Banos RM, Guillen V, Proctor C (2017). Positive technologies for improving health and well-being. Positive psychology interventions in practice.

[CR5] Bruner J (2004). Life as narrative. Social Research.

[CR6] Butler AC, Chapman JE, Forman EM, Beck AT (2006). The empirical status of cognitive–behavioral therapy: A review of meta-analyses. Clinical Psychology Review.

[CR7] Cooper P (2014). Using writing as therapy: Finding identity. The British Journal of Occupational Therapy.

[CR8] Elbert T, Schauer M (2002). Psychological trauma: Burnt into memory. Nature.

[CR9] Frankl VE (1969). The will to meaning: Foundations and applications of logotherapy.

[CR10] Fredrickson BL (2004). The broaden-and-build theory of positive emotions. Philosophical Transactions-Royal Society of London Series B Biological Sciences.

[CR12] Gerger H, Werner CP, Gaab J, Cuijpers P (2021). Comparative efficacy and acceptability of expressive writing treatments compared with psychotherapy, other writing treatments, and waiting list control for adult trauma survivors: A systematic review and network meta-analysis. Psychological Medicine.

[CR13] Glück J, Bluck S, Baron J, McAdams DP (2005). The wisdom of experience: Autobiographical narratives across adulthood. International Journal of Behavioral Development.

[CR15] González-Robles A, Suso-Ribera C, Díaz-García A, García-Palacios A, López DC, Botella C (2021). Predicting response to transdiagnostic iCBT for emotional disorders from patient and therapist involvement. Internet Interventions.

[CR16] Gordon KC, Baucom DH, Snyder DK (2004). An integrative intervention for promoting recovery from extramarital affairs. Journal of Marital and Family Therapy.

[CR17] Gortner EM, Rude SS, Pennebaker JW (2006). Benefits of expressive writing in lowering rumination and depressive symptoms. Behavior Therapy.

[CR18] Hayes SC (2004). Acceptance and commitment therapy, relational frame theory, and the third wave of behavioral and cognitive therapies. Behavior Therapy.

[CR20] Hull TD, Mahan K (2017). A study of asynchronous mobile-enabled SMS text psychotherapy. Telemedicine and e-Health.

[CR21] Hunt C (2010). Therapeutic effects of writing fictional autobiography. Life Writing.

[CR22] Jacinto GA, Edwards BL (2011). Therapeutic stages of forgiveness and self-forgiveness. Journal of Human Behavior in the Social Environment.

[CR23] Jans-Beken L, Jacobs N, Janssens M, Peeters S, Reijnders J, Lechner L, Lataster J (2020). Gratitude and health: An updated review. The Journal of Positive Psychology.

[CR24] Joseph S (2015). Positive therapy: Building bridges between positive psychology and person-centred therapy.

[CR25] Kaczmarek LD, Kashdan TB, Drążkowski D, Enko J, Kosakowski M, Szäefer A, Bujacz A (2015). Why do people prefer gratitude journaling over gratitude letters? The influence of individual differences in motivation and personality on web-based interventions. Personality and Individual Differences.

[CR26] King BR, Boswell JF (2019). Therapeutic strategies and techniques in early cognitive–behavioral therapy. Psychotherapy.

[CR27] King LA (2001). The health benefits of writing about life goals. Personality and Social Psychology Bulletin.

[CR28] Lambert NM, Clark MS, Durtschi J, Fincham FD, Graham SM (2010). Benefits of expressing gratitude expressing gratitude to a partner changes one’s view of the relationship. Psychological Science..

[CR29] Layous K, Lee H, Choi I, Lyubomirsky S (2013). Culture matters when designing a successful happiness-increasing activity: A comparison of the United States and South Korea. Journal of Cross-Cultural Psychology.

[CR30] Lichter I, Mooney J, Boyd M (1993). Biography as therapy. Palliative Medicine.

[CR31] Loveday PM, Lovell GP, Jones CM (2018). The best possible selves intervention: A review of the literature to evaluate efficacy and guide future research. Journal of Happiness Studies.

[CR32] Lyubomirsky S, Layous K (2013). How do simple positive activities increase well-being?. Current Directions in Psychological Science.

[CR33] Lyubomirsky S, Sousa L, Dickerhoof R (2006). The costs and benefits of writing, talking, and thinking about life's triumphs and defeats. Journal of Personality and Social Psychology.

[CR34] Manfrida G, Albertini V, Eisenberg E (2017). Connected: Recommendations and techniques in order to employ internet tools for the enhancement of online therapeutic relationships. Experiences from Italy. Contemporary Family Therapy.

[CR36] Martin RC, Dahlen ER (2005). Cognitive emotion regulation in the prediction of depression, anxiety, stress, and anger. Personality and Individual Differences.

[CR37] Masoni L (2019). Tale, performance, and culture in EFL storytelling with young learners: Stories meant to be told.

[CR38] McAdams DP, John OP, Robins RW, Pervin LA (2008). Life story: The encyclopedia of adulthood and aging. Handbook of personality: Theory & research.

[CR39] McAdams DP, Bauer JJ, Sakaeda AR, Anyidoho NA, Machado MA, Magrino-Failla K, Pals JL (2006). Continuity and change in the life story: A longitudinal study of autobiographical memories in emerging adulthood. Journal of Personality.

[CR40] McCullough ME, Root LM, Cohen AD (2006). Writing about the benefits of an interpersonal transgression facilitates forgiveness. Journal of Consulting and Clinical Psychology.

[CR42] Nicholls S (2009). Beyond expressive writing evolving models of developmental creative writing. Journal of Health Psychology.

[CR44] Pennebaker JW, Chung CK, Friedman H, Silver R (2007). Expressive writing, emotional upheavals, and health. Foundations of health psychology.

[CR45] Pennebaker JW, Evans JF (2014). Expressive writing: Words that heal.

[CR47] Pennebaker JW (2018). Expressive writing in psychological science. Perspectives on Psychological Science.

[CR48] Pennebaker JW, Beall SK (1986). Confronting a traumatic event: Toward an understanding of inhibition and disease. Journal of Abnormal Psychology.

[CR49] Pennebaker JW, Boyd RL, Jordan K, Blackburn K (2015). The development and psychometric properties of LIWC2015.

[CR50] Pennebaker JW, Mehl MR, Niederhoffer KG (2003). Psychological aspects of natural language use: Our words, our selves. Annual Review of Psychology.

[CR51] Rash JA, Matsuba MK, Prkachin KM (2011). Gratitude and well-being: Who benefits the most from a gratitude intervention?. Applied Psychology: Health and Well-Being.

[CR52] Rashid T (2015). Positive psychotherapy: A strength-based approach. The Journal of Positive Psychology.

[CR53] Rashid T, Seligman MP (2018). Positive psychotherapy: Clinician manual.

[CR54] Reese HW (2013). The perception of stimulus relations: Discrimination learning and transposition.

[CR55] Reinhold M, Bürkner PC, Holling H (2018). Effects of expressive writing on depressive symptoms—A meta-analysis.

[CR56] Reker GT, Birren JE, Svensson C (2014). Self-aspect reconstruction through guided autobiography: Exploring underlying processes. The International Journal of Reminiscence and Life Review.

[CR57] Ruini C, Ottolini F (2014). The use of narrative strategies based on fairytales as a novel, integrative ingredient in CBT: A case report. EXPLORE: The Journal of Science and Healing.

[CR58] Ruini C, Albieri E, Ottolini F, Vescovelli F (2020). Once upon a time: A school positive narrative intervention for promoting well-being and creativity in elementary school children. Psychology of Aesthetics, Creativity, and the Arts.

[CR59] Ruini C (2014). The use of well-being therapy in clinical settings. The Journal of Happiness & Well-Being.

[CR60] Ruini C (2017). Positive psychology in the clinical domains: Research and practice.

[CR61] Sandstrom MJ, Cramer P (2003). Defense mechanisms and psychological adjustment in childhood. The Journal of Nervous and Mental Disease.

[CR62] Sauter T (2013). ‘What’s on your mind?’ Writing on Facebook as a tool for self-formation. New Media & Society.

[CR64] Seih YT, Chung CK, Pennebaker JW (2011). Experimental manipulations of perspective taking and perspective switching in expressive writing. Cognition & Emotion.

[CR65] Sheldon KM, Lyubomirsky S (2006). How to increase and sustain positive emotion: The effects of expressing gratitude and visualizing best possible selves. The Journal of Positive Psychology.

[CR66] Shin LJ, Armenta CN, Kamble SV, Chang SL, Wu HY, Lyubomirsky S (2020). Gratitude in collectivist and individualist cultures. The Journal of Positive Psychology.

[CR67] Slatcher RB, Pennebaker JW (2006). How do I love thee? Let me count the words: The social effects of expressive writing. Psychological Science.

[CR70] Sloan DM, Sawyer AT, Lowmaster SE, Wernick J, Marx BP (2015). Efficacy of narrative writing as an intervention for PTSD: Does the evidence support its use?. Journal of Contemporary Psychotherapy.

[CR71] Smyth JM (1998). Written emotional expression: Effect sizes, outcome types, and moderating variables. Journal of Consulting and Clinical Psychology.

[CR72] Snyder CR, Rand KL, Sigmon DR, Snyder CR, Lopez SJ (2002). Hope theory. Handbook of positive psychology.

[CR73] Snyder CR, Ilardi SS, Cheavens J, Michael ST, Yamhure L, Sympson S (2000). The role of hope in cognitive-behavior therapies. Cognitive Therapy and Research.

[CR74] Sorapure M (2003). Screening moments, scrolling lives: Diary writing on the web. Biography.

[CR75] Staudinger UM (2008). A psychology of wisdom: History and recent developments. Research in Human Development.

[CR76] Taylor L, McMinn MR, Bufford RK, Chang KB (2010). Psychologists’ attitudes and ethical concerns regarding the use of social networking web sites. Professional Psychology: Research and Practice.

[CR77] Thoresen CE, Luskin F, Harris AH (2008). Science and forgiveness interventions: Reflections and recommendations. Dimensions of Forgiveness: A Research Approach.

[CR79] Toepfer SM, Cichy K, Peters P (2012). Letters of gratitude: Further evidence for author benefits. Journal of Happiness Studies.

[CR80] Van Deurzen E (2012). Existential counselling & psychotherapy in practice.

[CR83] Wong YJ, Owen J, Gabana NT, Brown JW, McInnis S, Toth P, Gilman L (2018). Does gratitude writing improve the mental health of psychotherapy clients? Evidence from a randomized controlled trial. Psychotherapy Research.

[CR84] Wright J, Chung MC (2001). Mastery or mystery? Therapeutic writing: A review of the literature. British Journal of Guidance and Counselling.

[CR85] Yalom ID (1980). Existential psychotherapy.

